# Authors' Response to Hockey and Reidak

**DOI:** 10.1371/journal.pmed.0030407

**Published:** 2006-09-26

**Authors:** Julio Licinio

Because the increased prescriptions of antidepressants are correlated to increased medical visits, it is tempting to conclude, as Hockey did [1], that decreased suicides are a function of greater recognition of depression. It should be noted that the biggest cause of suicide is clinical major depression and increased visits do not treat that; antidepressants do.

In a comprehensive review of the literature on the role of long-term antidepressant use to prevent relapse of major depression, Geddes et al. [2] reported that “data were pooled from 31 randomised trials (4410 participants). Continuing treatment with antidepressants reduced the odds of relapse by 70% (95% CI 62–78; 2p<0.00001) compared with treatment discontinuation. The average rate of relapse on placebo was 41% compared with 18% on active treatment”. We therefore conclude that just seeing a doctor is in the long term not protective against major depression and its consequences, such as suicide. The weight of existing data supports a positive effect of antidepressants. It is plausible that effective long-term treatment of depression by other methods might also be beneficial.

In response to the query from Reidak regarding Eli Lilly, I must say that I completely disclose all my activities, and that is why Aasa Reidak was able to write her letter [3]. I had published before on this topic in *Nature Reviews Drug Discovery* and had data (which were widely known to all in the field, including Eli Lilly) that since fluoxetine was introduced, prescriptions had gone up and suicide rates had gone down. There is nothing really conceptually new there. That was what was presented at one of Eli Lilly’s regular weekly scientific sessions, which exist at most research institutions, including Lilly Research Laboratories. The paper published here is on the modelling of suicide rates using pre-1988 data to estimate what suicide rates would be now and therefore to predict a potential putative effect of fluoxetine and other selective serotonin reuptake inhibitors [4] . These mathematical modelling data are new to this paper, and that entire analysis and manuscript content took place without the knowledge, support, or input of Eli Lilly.

The work reported in the article was done in the absence of any conflict of interest or pharmaceutical industry support. After the paper was submitted for publication in *PLoS Medicine*, I agreed to provide consultations for Eli Lilly, the manufacturer of fluoxetine. This has been a minor, occasional role, with very limited compensation. Such a relationship did not exist and was not planned when the work was done or the article written and submitted, and it is being disclosed here in the interests of transparency.
